# Exploring the care provided to mothers and children by community health workers in South Africa: missed opportunities to provide comprehensive care

**DOI:** 10.1186/s12889-018-5056-y

**Published:** 2018-01-23

**Authors:** A. Wilford, S. Phakathi, L. Haskins, N. A. Jama, N. Mntambo, C. Horwood

**Affiliations:** Centre for Rural Health, University of KwaZulu-Natal, 4th Floor George Campbell Building, Howard College campus, Durban, 4041 South Africa

**Keywords:** community health workers, maternal health, child health, South Africa

## Abstract

**Background:**

Community health workers (CHWs) provide maternal and child health services to communities in many low and middle-income countries, including South Africa (SA). CHWs can improve access to important health interventions for isolated and vulnerable communities. In this study we explored the performance of CHWs providing maternal and child health services at household level and the quality of the CHW-mother interaction.

**Methods:**

A qualitative study design was employed using observations and in-depth interviews to explore the content of household interactions, and experiences and perceptions of mothers and CHWs. Fifteen CHWs and 30 mothers/pregnant women were purposively selected in three rural districts of KwaZulu-Natal, SA. CHW household visits to mothers were observed and field notes taken, followed by in-depth interviews with mothers and CHWs. Observations and interviews were audio-recorded. We performed thematic analysis on transcribed discussions, and content analysis on observational data.

**Results:**

CHWs provided appropriate and correct health information but there were important gaps in the content provided. Mothers expressed satisfaction with CHW visits and appreciation that CHWs understood their life experiences and therefore provided advice and support that was relevant and accessible. CHWs expressed concern that they did not have the knowledge required to undertake all activities in the household, and requested training and support from supervisors during household visits.

**Conclusions:**

Key building blocks for a successful CHW programme are in place to provide services for mothers and children in households but further training and supervision is required if the gaps in CHW knowledge and skills are to be filled.

## Background

Community health workers (CHWs) are frequently deployed as a component of the health system to provide health services to women and children in their homes, particularly in low and middle-income countries where professional health workers are scarce. CHWs are broadly defined as members of a community, who are chosen by their community and work within their community to provide culturally appropriate health services [1]. CHWs are supported by the health system but have no professional training [2], and are usually volunteers or receive a stipend for their work. Their role may include provision of preventive, promotive as well as curative services.

CHWs can improve access to care and coverage of vital maternal and child health (MCH) services to women and children in communities, and in some settings this has been shown to improve child mortality [3–6], CHWs can provide continuity of care from the antenatal to the postnatal period, providing women with accessible, appropriate advice on pregnancy, childbirth and postnatal care, as well as HIV prevention and treatment practises, and improving the linkages between communities and formal health services.

Despite recent improvements in child mortality, reducing child and maternal morbidity and mortality remains a priority in South Africa (SA). If this is to be achieved, coverage of key interventions must be improved [7], particularly early access to antenatal care, improved coverage of postnatal care, access to infant feeding support, and prevention of mother-to-child transmission of HIV (PMTCT) interventions. Current guidelines for PMTCT recommend that all HIV-infected pregnant women start lifelong ART at the time of HIV diagnosis, and mothers should continue to breastfeed their infants for up to 24 months as long as they are adherent to ART [8]. Retention in care for HIV-infected mothers and their children is crucial for improving health outcomes in high HIV prevalence settings like SA. There is also a need to build additional capacity for delivery of cost-effective interventions to poor and vulnerable communities, while recognising the extreme shortage of health professionals in underserved areas. Delayed access to antenatal and postnatal care has been reported as one of the causes of maternal and child deaths [9] and many child deaths occur at home [10].

CHWs are widely deployed in South Africa, particularly in underserved areas. Recognition that provision of home and community-based health services, together with strong linkages to health facilities, is key to improving health outcomes [11] has led to deployment of increasing numbers of CHWs in SA in recent years as part of implementation of the Re-engineering of Primary Health Care policy [12]. CHWs are allocated a number of households that they serve and are expected to visit regularly (3–5 households per day). During household visits they perform a variety of functions, which include treatment support and home-based care, as well as MCH activities. Core functions for MCH include visiting all mothers during pregnancy and in the postnatal period to provide education and support in several key areas including antenatal care attendance, planning for delivery of the baby, postnatal care and support for infant feeding. In SA, CHWs undergo two weeks training in maternal and child health care as part of their overall training, and receive a small stipend. However, little is known about the quality of the care provided at household level.

This study aimed to explore the quality of CHW household visits to mothers and children using observations and in-depth interviews with mothers and CHWs. As far as we are aware, this is the first study in South Africa to directly observe and assess care provided by CHWs in households.

## Methods

### Study area

This study was conducted in three predominantly rural districts of the eleven health districts in KwaZulu-Natal (KZN), SA. The three districts were purposively selected by the KZN Department of Health based on availability of CHWs working in ward-based outreach teams in these areas.

The three districts serve a population in excess of 2.3 million inhabitants. Health care is provided through the district health system, based on a primary health approach. In the study areas CHWs worked within a ward-based outreach team, and were supervised by a community-based registered nurse. CHWs are linked to their local PHC clinic, where they receive referrals and to which they refer when necessary.

### Study design

**Fig. 1:**
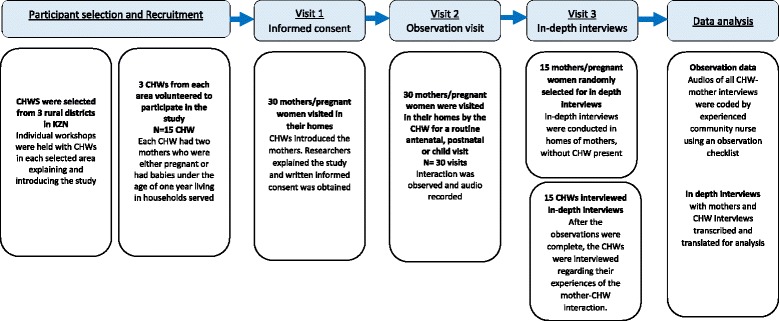
Flow diagram of participants included in the study

This study employed a qualitative design to explore the quality of MCH services and health promotion messages provided to women and infants by CHWs visiting households. A CHW visit to a mother or pregnant woman was observed by a field worker, followed by an in-depth interview with the participating women and CHWs. Observations and interviews were collected over a series of visits to each community and each household (Fig. 1).

### Selection of study participants

Five communities were selected in the three districts in partnership with the KZN Department of Health (DoH), using a convenience sample based on availability of CHWs in these areas. CHWs were approached by researchers and informed about the study. CHWs were selected based on their willingness to participate and on their having two eligible women residing in the households they served. Women were eligible to participate if they were pregnant or had babies under the age of one year, and were residing in households visited by participating CHWs. Three CHWs from each community were selected to participate. Thus, 15 CHWs and 30 women were included in the study. Of the 30 women selected for observation, 10 were pregnant and 20 had delivered their baby, eight of whom had a baby younger than 28 days. As a result, 10 mothers received antenatal visits, 8 mothers received postnatal visits and 12 mothers received child visits.

### Data collection

All household visits and interviews were conducted in isiZulu. A researcher observed the household visit undertaken by the CHW, and all verbal interactions between the CHW and mother during the visit were audio recorded [13, 14]. During the household visit, the researcher took field notes to record the activities undertaken during the visit that may not be identified from the audio-recording, for example non-verbal communication and clinical examination. In-depth interviews were conducted with participating women and CHWs at a later visit to explore perceptions, experiences and attitudes about the CHW visit, the context in which CHWs operate, their perceived competencies, and the support and supervision given to CHWs (Fig. 1). All interviews were audio recorded.

A content checklist was developed to assess the quality of the visit, including the information provided and activities undertaken. This checklist was based on the SA DoH policy for community-based health interventions for mothers and children and the training that CHWs received in caring for mothers and infants in the household [13]. These documents set out clearly the expected activities of CHWs during household visits.

### Data analysis

Audio-recordings of the CHW- mother interaction were coded for content by a registered nurse using the relevant content checklist for the type of interaction (antenatal, postnatal or child visit).

All interviews were transcribed verbatim, translated into English and exported to NVivo version 10. Thematic content analysis was performed on the interview transcripts. The themes that emerged from the analysis of the transcripts were reviewed independently by two researchers, and were then triangulated with the content analysis coding of the observation data. Field notes were used during the analysis to contextualise the content analysis of the household visit and the interview findings.

## Results

Observations and interviews were conducted between November 2014 and January 2015. Demographic information about participating mothers is shown in Table 1.

**Table 1:** Demographic information about participants

*N* = 30	n
Mother category	
Pregnant mother	10
Post-natal visit (before 28 days post-partum)	8
Child visit (age from 28 days to < one year)	12
First CHW visit for this mother	
Yes	9
No	21
Antenatal visit - Gestational age (*N* = 10)	
3-4 months	2
5–6 months	3
7–8 months	5
	Average
Post-natal visit Child age (*N* = 8)^a^	1.5 weeks
Child visit Child age (*N* = 12)^a^	5.5 months

^a^1 missing

Observations showed that during most household visits CHWs introduced the visit appropriately (29/30; 97%) and mothers were given the opportunity to ask questions (23/30; 77%). CHWs encouraged mothers to talk (24/30; 80%) and responded appropriately to questions (23/30; 77%). During some visits the CHWs checked the mothers understanding of advice given (16/30; 53%), and made a plan for the way forward (17/30; 57%).

**Fig. 2:**
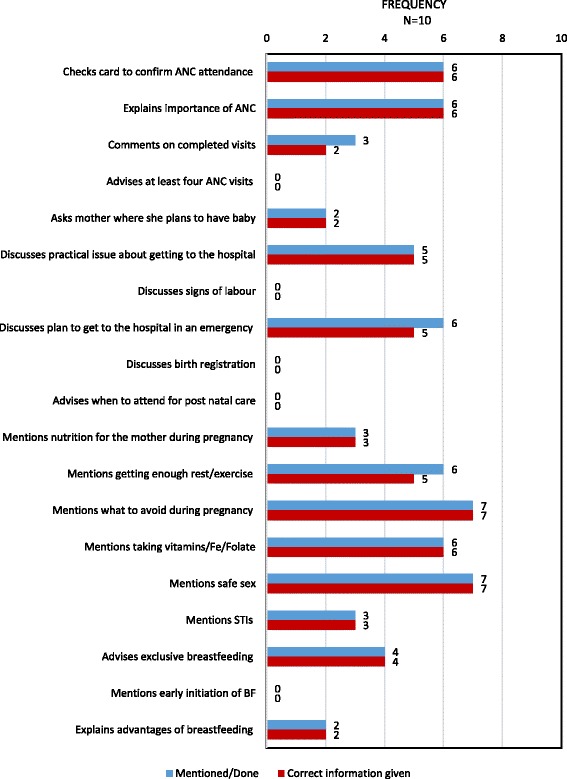
Topics discussed by CHWs during antenatal home visits

**Fig. 3:**
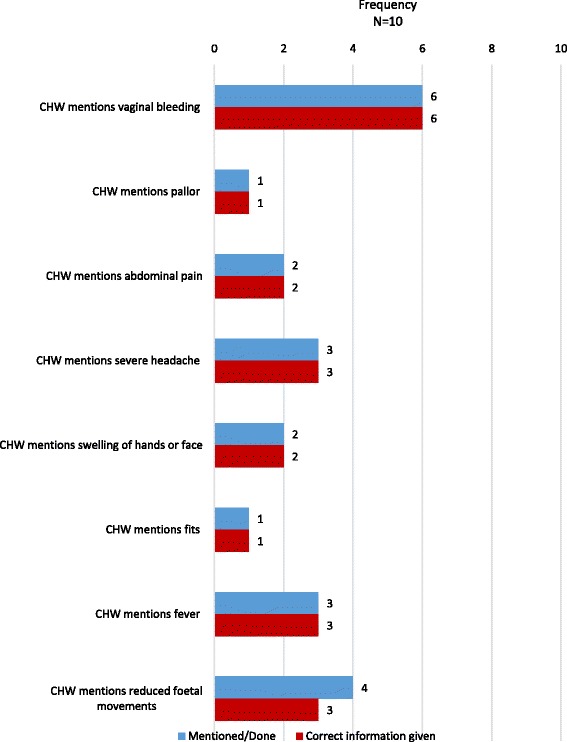
Pregnancy related danger signs discussed by CHWs during antenatal home visit

Topics discussed by CHWs with mothers during antenatal observations are shown in fig. 2. Gaps in the health messages provided by CHWs were noted around planning for the delivery of the baby, breastfeeding and postnatal care. Danger signs in pregnancy were incompletely covered or not mentioned at all (fig. 3). However, when information was provided, it was usually correct, with few instances of incorrect or inappropriate information being provided to mothers.

**Fig. 4:**
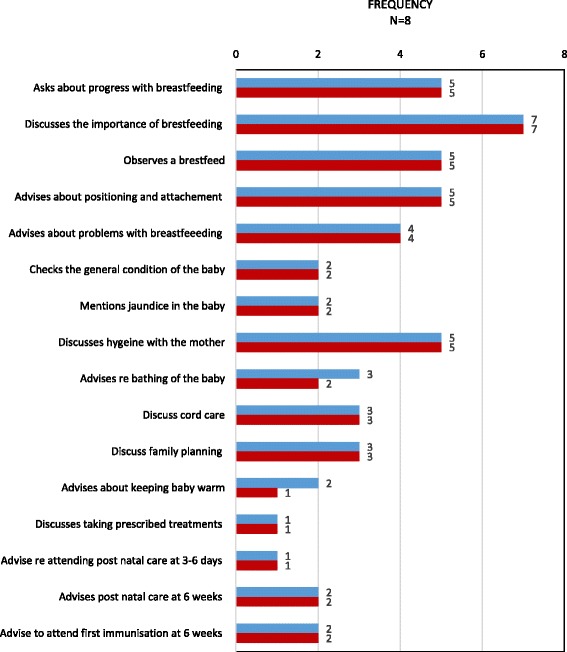
Topics discussed by CHWs during a postnatal home visit

**Fig. 5:**
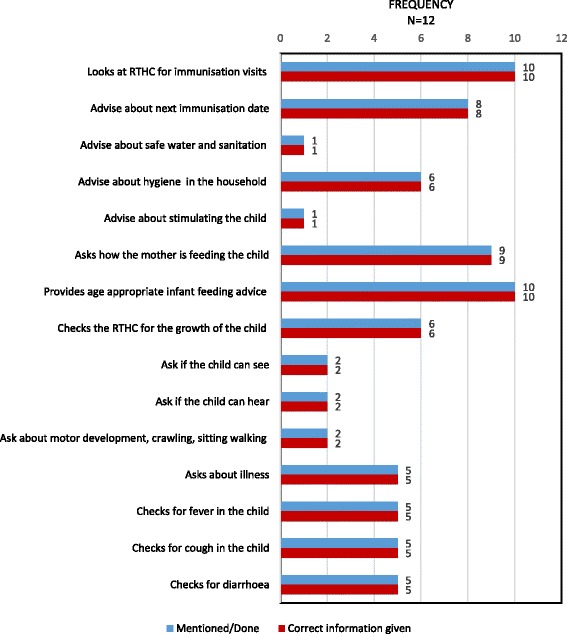
Topics discussed by CHW while visiting a mother with an older child

Similarly, although good quality information was provided during postnatal and child visits, several key message were not mentioned, including key aspects of home care for newborn babies (fig. 4) and older children (fig. 5).

The observations demonstrated that in some cases CHWs did not respond to mothers’ questions regarding their own health and that of their babies. One mother expressed concern about her baby’s health saying the cord was bothering the baby, another mother mentioned that her baby was coughing, and a third mother said that she was concerned about her baby’s eyes and that she herself was still experiencing vaginal bleeding. In each of these cases the baby and/or mother were not examined, the mothers’ questions were not addressed and the mother and/or child were not appropriately referred. Furthermore, the observer noted during postnatal and child visits CHWs did not examine mothers and babies for signs of illness.

### In-depth interviews with CHWs

One of the strongest themes emerging from the CHW interviews was the CHWs perception that they had inadequate knowledge on some topics which undermined their performance during household visits*.* CHWs were aware of this and explained that at times they were unable to respond to mothers’ questions.

*‘I do have knowledge but it is not adequate. Perhaps I need to be given additional information. There are questions that they ask where you find that I will not be confident when I respond to them.’* **CHW 09 District 03.**

This lack of knowledge and skills also led to CHWs being reluctant to examine the mothers and infants.

*‘When we go to check a newborn child, we do not have a clue about what are we going to check. We just get there and look at the child and see that they are able to feed.’* **CHW 14 District 02.**

CHWs suggested that they should receive additional training to ensure that their skills and knowledge were improved and updated.

*‘Information has to be updated regularly through workshops because sometimes after many years you forget things and these government things change all the time. They do not remain static’* **CHW 03 District 01.**

CHWs unanimously expressed the need for a supervisor to assist them, saying that they felt motivated and encouraged by the supervision.

*“I like being supervised because it motivates you in your work”* **CHW15 District 02.**

*“Having other people come with you when you visit households encourages us…It also gives us that support.*” **CHW 13 District 02.**

Some CHWs suggested that supervisors accompany them during their household visits to provide support, as this would also help to close the gaps between their knowledge and training, as well as to giving them more credibility in the eyes of the community.

*“I like being supervised because people are used to me but they are not used to my supervisor. If I am with her, let us say a person is stubborn and does not want to listen to me, when I visit them with my supervisor and tell them that I am with my senior and ask that they speak to my supervisor because they do not want to listen to me. My supervisor then speaks to them and the situation ends up changing.*” **CHW 14 District 02.**

However, CHWs expressed that they usually have to wait for their next supervisor meeting to raise concerns about their visits, but CHWs also acknowledged that their supervisors faced challenges preventing them from supervising more frequently. In particular, supervisors had a large number of CHWs to oversee.

*“She (CHW supervisor) had never gone to the field with me…you find that you do not get assistance with certain things that you need to be assisted in when you visit homes. You end up having to wait for the next meeting at the clinic, and that is the only time you can ask about things that were challenging to you when you were trying to educate the family.”* **CHW 09 District 03**.

CHWs also frequently identified lack of tools and equipment such as MUAC tapes and thermometers as a challenge when visiting the households, and even when they had access to these tools, they were not trained in their use. This could undermine their ability to function effectively in the household.

*“I think that is also a problem because when we visit a household we do not have tools of trade, we do not have enough and we are not trained on how to use them.”* **CHW 13 District 02**.

### In depth interviews with mothers

All mothers strongly expressed their appreciation of the CHW visits. During the interviews mothers shared many stories of how CHWs helped them during their pregnancy and with the newborn baby.

*‘What I liked is that she was able to give me advice and I listened to her. It helped me and she was complimented at the clinic as well. They asked what brought me to the clinic. I told them it was the CHW who said I must come to the clinic. They said she has done well and I went to the clinic early during my pregnancy.’* **Mother 03 District 01.**

*‘Yes it has been very helpful because I look at some of the things that she [CHW] says. I get in touch with her all the time because she is someone who is lives nearby. Ever since she started it has been very helpful to my children.’* **Mother 20 District 01.**

Many of the women expressed that they had some concerns about disclosure of private information when the CHW started visiting the household, but as time went on they were reassured that the CHW did not gossip about others, and they were able to disclose private information.

*‘You will not hear another person talking about your problem. It remains between the two of you. She just keeps asking how you feel and how things are going. You then tell her that here it is better, there it is still not fine. She gives you good advice and she is very patient. She gives you your time. She is very helpful’* **Mother 07 District 01.**

Mothers expressed appreciation that CHWs were trained to provide care and were able to provide information in a way that they could easily understand. In particular, mothers appreciated that the CHW is close by and could be called quickly in an emergency, and that CHWs were from their own community and were able to understand the lived experiences of the mothers.

‘*I am happy about that because I know it is someone who knows my condition, someone who knows where I come from. It is better to have someone who knows you who will be able to give you the right advice.’* **Mother 14 District 03.**

Mothers shared that they were generally satisfied with CHW visits to their home because it allowed sufficient time for them to discuss different topics which is not always possible in the clinic setting with a nurse.

*‘Even when you go to the clinic, there is not enough time to talk like the time the CHW has. During the [CHW] visits there is enough time to talk unlike the time at the clinic. I liked that because we receive more information than we get at the clinic because the time at the clinic is not enough, nurses are rushing to service everyone.’* **Mother 16 District 03.**

Several of the women had disclosed that they were HIV positive to their CHW. They spoke about how the CHW had encouraged them to visit the clinic early for HIV testing.

*‘She did a good thing because she is a CHW and it is her job to find out things about people in the community. As an HIV positive person she sat me down and told me to attend the antenatal clinic as soon as possible so I could get tested.’* **Mother 22 District 02.**

## Discussion

This study shows that CHWs visiting mothers and children in their homes are well accepted and appreciated by mothers. CHWs provided important appropriate health promotion messages, at a level that was understood by mothers. However, we identified missed opportunities to provide important information to mothers that could directly impact health outcomes for mothers and children, including information about danger signs in pregnancy, importance of postnatal care, checking the condition of the newborn baby, and checking development in older children. CHWs are aware of these shortfalls and identified lack of training, inadequate resources and poor supervision as limiting their ability to provide recommended care in the household.

Gaps identified suggest that training should include practical skills components, rather than only the classroom-based instruction currently available in South Africa. CHWs need practical examination skills to check the condition of the mother and baby in the days after delivery, and to identify danger signs. The early postnatal period is a vulnerable time for mothers and babies, with high morbidity and mortality [14], and early access to postnatal care is a key intervention to improve outcomes [15]. CHWs could provide a crucial link with formal health services during this period but require practical skills to examine mothers and babies and identify danger signs if they are to do so effectively. Supporting breastfeeding is another crucial, lifesaving intervention where CHWs can provide the practical help for mothers to initiate and sustain breastfeeding, and improve rates and duration of exclusive breastfeeding.

CHWs were observed failing to respond to concerns expressed by mothers regarding their health and that of their babies, and CHWs stated that they did not have the knowledge or competence to address them.

Although deployment of CHWs is a strategy employed in many countries to provide accessible care to mothers and babies in the household, little is known about the quality of CHW-mother interactions in the household and whether relevant activities and information are being provided. Despite the gaps identified, we rarely found that CHWs provided inappropriate or incorrect information to mothers. CHWs were able to engage with mothers positively and mothers were unanimous in expressing appreciation of these visits, citing that CHWs had more time compared to the clinic, that CHWs were trusted, accessible and able to understand the mothers’ situation. This strongly supports the CHW programme as having potential to provide MCH services in our setting.

CHWs work in a challenging environment where they have to navigate complex social contexts where there may be little privacy, and, as members of the community may have personal relationships with community members, as well as providing services and dealing with confidential information. Several mothers mentioned disclosure of sensitive information to CHWs as a concern, but mothers reported being reassured about confidentiality as visits progressed, and trust was built up over time. Consequently, CHWs were able to play a strong role in supporting HIV infected women within the family and household. Our findings suggest that CHWs are able to successfully achieve the balance between their roles as a community member and a CHW. However, it is important that CHWs receive guidance on issues of confidentiality and professionalism during training, as breaches of confidentiality could undermine the success of the CHW programme.

Our findings suggest that, although the foundations of a successful, effective community-based service were in place, there is potential to build on this success by improving knowledge, skills and self-efficacy among CHWs. CHWs were aware of the challenges, and felt that these inadequacies undermined their credibility in the eyes of the community. CHWs suggested that more training and supervision, particularly at household level could assist to address these challenges. Health managers implementing CHW programmes expect CHWs with little training to provide a broad range of services, often in isolated communities. Support is clearly required if such an approach is to be successful, and several studies have identified supervision as key to the success of CHWs programmes [16, 17]. Supervision also raises awareness of CHWs role, can legitimise the CHWs role in the community, as well as improving motivation and retention among CHWs [17]. Supervision and mentoring is essential for improving the quality of service delivery [18–20]. However, regular and sustained quality supervision and support, is uncommon in health services in low and middle-income settings, even for professional health workers [16, 21]. Supervision and support at community level, in particular, remains a considerable challenge, and has rarely been sustained at scale. To improve supervision, a number of models have been proposed for use with CHWs, which include peer supervision [16], telephonic access to supervisors [22], establishment of community supervisors [23], supportive supervision [24], self-assessment and a quality improvement approach [25]. However, many of these studies demonstrate improvement in the frequency of supervision rather than improvements in the quality of supervision. [16, 20].

In SA, the establishment of ward-based outreach teams (WBOTs) comprising registered nurses, enrolled nurses and CHWs who work together to provide community-based care provides an opportunity for developing a supervision model. However, WBOTs are costly, have been difficult to sustain and even more difficult to scale-up to provide universal coverage. It may be inappropriate to use registered nurses to provide supervision for large numbers of CHWs, for both cost and resource reasons, as proposed in this approach. More research is required to establish whether this is an effective, affordable and sustainable model for providing support and supervision of CHWs.

We employed a strong study design using field notes, audio-recordings and in-depth interviews to collect data to contextualise our findings and to explore different dimensions and perspectives of the CHW-mother interaction. Our approach also allowed for triangulation of data from different sources thus improving the validity of the data. However, several limitations were anticipated in the study design. Three rural districts were included in this research, thus findings are not generalizable to the whole province. However, many South Africans live in similar rural areas and face similar daily challenges. In addition, the presence of the observer is likely to have affected the communication between the mother and CHW during the visit. However, several actions were taken to limit the observer effect, namely the observer was of similar age and race as the CHW; there was a separate visit to obtain informed consent before the observation; the researcher did not participate in the household visit, but sat quietly and unobtrusively away from the CHW and mother. Transcribing and translating of interviews conducted in isiZulu may have led to some loss of information. Some topics that appear to have been omitted during the visit may have been addressed on previous visits.

## Conclusions

This study suggests that although CHWs are well accepted and appreciated by the mothers they visit, the care they provide is sub-optimal with many missed opportunities to provide important health information and to identify important health issues in the household. A comprehensive and sustainable package of skills development, support and supervision of CHWs is required if this cadre is to reach their full potential and provide effective care for mothers and babies in the community.

## Abbreviations

ART: Anti-Retroviral Therapy
CHW: Community Health Worker
DoH: Department of Health
HIV: Human Immuno Virus
KZN: KwaZulu-Natal
MCH: Maternal and Child Health
PMTCT: Prevention of Mother to Child Transmission
SA: South Africa
WBOT: Ward Based Outreach Team
